# Utilization of apnea-hypopnea index as a novel predictive factor for difficult mask ventilation in the Chinese population under general anesthesia

**DOI:** 10.1007/s11325-021-02310-6

**Published:** 2021-02-06

**Authors:** Jiayi Wang, Jingjie Li, Pengcheng Zhao, Xuan Pu, Rong Hu, Hong Jiang

**Affiliations:** 1 Department of Anesthesiology, Shanghai Ninth People’s Hospital, Shanghai JiaoTong University School of Medicine, Zhizaoju Road, 639, Shanghai, 200011 China; 2 Departments of Outcomes Research, Cleveland Clinic, Cleveland, OH USA; 3 Quantitative Health Sciences, Cleveland Clinic, Cleveland, OH USA

**Keywords:** Difficult airway management, Difficult mask ventilation, Apnea-hypopnea index

## Abstract

**Purpose:**

Difficult mask ventilation (DMV) is a potentially life-threatening situation that can arise during anesthesia. However, most clinical predictors of DMV are based on European and US populations. On the other hand, most predictive models consist of multiple factors and complicated assessments. Since obstructive sleep apnea (OSA) is among the most important risk factors associated with DMV, the apnea-hypopnea index (AHI) may play an important role in determining patient risk.The purpose of this study was to investigate the relationship between DMV and AHI, and to determine preoperative risk factors for DMV in Chinese patients.

**Methods:**

A prospective cohort trial enrolled patients scheduled for elective surgery. After obtaining informed consent, patient demographic information was collected, and patients were tested with pre-operative polysomnography. The anesthesiologist who managed the airway graded the mask ventilation. The difficult mask ventilation was defined as the mask ventilation provided by an unassisted anesthesiologist without oral airway or other adjuvant. A logistic regression model was used to analyze the association between AHI and DMV.

**Results:**

A total of 159 patients were analyzed. For both primary and secondary outcomes, the unadjusted and adjusted odds ratio for DMV showed significant increases by 5 AHI units. AHI, age, and the Mallampati classification were found to be independent predictive factors for DMV.

**Conclusions:**

AHI is associated with DMV as a novel independent risk factor in Chinese patients. Along with age and Mallampati classification, AHI should be included in establishing a superior predictive strategy for DMV screening.

**Trial registration:**

Chinese Clinical Trial Registry ChiCTR-DDD-17013076

## Background

One essential aspect of airway management in general anesthesia is to evaluate the effectiveness of mask ventilation for patients [1]. Among all cases, the incidence of difficult mask ventilation (DMV) is between 0.08 and 15% [2–5]. Difficult ventilation is considered the major contributing factor for morbidity and mortality related to anesthesia [1, 6]. It is important to evaluate the airway and predict the problems prior to induction; the 4th National Audit Project (NAP4) and other major anesthesia societies recommend a pre-operative assessment for every patient’s airway [1, 7–9]. The American Society of Anesthesiologists (ASA) defines DMV as a situation where it is not possible for an unassisted anesthesiologist (1) to maintain an oxygen saturation > 90% using 100% oxygen and positive pressure ventilation, or (2) to prevent or reverse signs of inadequate ventilation [1]. Over the years, a series of studies on the prediction and treatment of DMV (difficult mask ventilation) have been conducted. An age >55, body mass index (BMI)>26 kg/m^2^, presence of beard, no teeth, and snoring are five independent factors related to difficult mask ventilation [3]. In addition, neck radiotherapy, neck circumference, male gender, Mallampati grade, and severely limited jaw protrusion are known as risk factors for DMV [2, 4, 10].

However, most of the above predictors remain unable to fully evaluate DMV risk. One important reason is that most clinical predictors are based on European and US populations. To contrast this, risk assessment for DMV in Chinese populations is complicated by the relatively limited number of studies with these geographic and ethnic populations. Moreover, there are discernable differences between Western and Asian populations; fewer bearded patients can be found in the Chinese population. Other differences between the populations include variations in general head and facial structures. Some of these predictors, such as age, BMI, neck circumference, male, and limited jaw protrusion, are closely related to patients who have obstructive sleep apnea (OSA).

It is well-established that a history of OSA is an independent predictor of impossible mask ventilation [3]. Polysomnography (PSG) is the diagnostic standard of OSA [11]. With the advancement of technology for PSG, devices are becoming more portable and easy for patients to wear. One important parameter from PSG is the apnea-hypopnea index (AHI), which is calculated as the sum of all apneas and hypopneas, divided by total hours of sleep time. An AHI of 15 or more events per hour, or five or more events per hour in the presence of symptoms or cardiovascular comorbidities, is diagnosable for OSA [11, 12].

Furthermore, most predictive models of DMV evaluation consist of multiple factors and complicated assessments which are time-consuming [2, 13]. Among these models, there are factors which base on patient’s subjective memory such as snoring history and alcoholism history.

With limited evidence on the predictors in the Chinese population and a close connection between OSA and DMV, we hypothesized that AHI is a predictive factor of DMV in the Chinese population. Secondly, we attempted to discover the predictors which are more appropriate for the Chinese population.

## Methods

### Ethical statement

IRB approval by the Ethics Committee (IRB No. 62017-362-T264) was obtained in September of 2017. A study population of 200 patients older than 18 years old underwent elective surgery, with ASA I-III, and general anesthesia over a 12-month period (October 2018–October 2019). Patients with obvious airway malformations, patients undergoing regional anesthesia, and those with contraindication of mask ventilation (i.e., planned awake intubation) were excluded from the study. All patients provided informed consented prior to surgery.

### Measurements

During the preoperative visit, the following information was collected by two anesthesiologists: age, gender, height, weight, BMI, the presence of facial beard, no teeth, neck circumference, history of snoring, modified Mallampati classification (modified by Samsoon and Young [14], performed with the patient in the sitting position with the head in full extension, tongue out, and with phonation), thyromental distance (measured with the patient in sitting position and head in extension[15]), ability to extend lower jaw, alcohol addiction, history of neck radiotherapy, history of DMV and difficult intubation (DI), and past medical and surgical history.

All patients were scheduled for over-night polysomnography (PSG). All PSGs were performed using a home sleep testing (HST) device (Alice NightOne, Philips, USA). PSG consisted of continuous recording with three sensors (respiratory effort belt, nasal cannula, and non-invasive pulse oximetry). PSGs were scheduled to last between 6 and 8 h and terminated following the final waking. All PSG studies were scored by a registered PSG technician. All studies were reviewed and interpreted by a physician board-certified in sleep medicine. AHI was obtained from the PSG reports.

During induction of anesthesia, a firm pad was placed under the patient’s skull and the head was extended by the neck (“sniffing position”). Preoxygenation of each patient lasted 4 min by mask (Flexicare, UK; size 3, 4) with 100% O_2_. Each patient was routinely monitored during the whole procedure by electrocardiography, non-invasive blood pressure, oxygen saturation (SpO_2_), and end-tidal carbon dioxide tension (Datex-Ohmeda Avance, GE, USA). The same anesthesiologist who obtained information during preoperative assessments also managed the airway, but was blinded to the PSG results. The anesthesiologist graded the mask ventilation as either easy or difficult. In this study, the difficult mask ventilation was defined as the mask ventilation provided by unassisted anesthesiologist without oral airway or other adjuvant according to Langeron et al. [3].

### Statistical analysis

AHI was assumed to be left-skewed and approximately followed a lognormal distribution, with a location parameter of 2 and scale parameter of 1[16]. Based on previous observations, the incidence of difficult group was about 30%. A total of 200 patients would have 90% power to detect a clinically important odds ratio of 1.25 associated with a 5-unit increase in AHI.

We summarized the baseline characteristics using standard statistics. Continuous variables with normal distribution were reported as mean ± standard deviation. Non-normal continuous variables were reported as median (interquartile range). Categorical variables were reported as frequency (percentage).

A logistic regression model was used for the primary analysis to assess the association between AHI and DMV, without adjusting other variables. Crude odds ratio and a 95% confidence interval was reported. A *p*-value <0.05 was considered significant.

The correlation between AHI and other variables, including age, gender, BMI, neck circumference, modified Mallampati classification, thyromental distance, ability to extend lower jaw, and alcoholism, was also explored. Pair-wise Pearson correlation coefficients were reported. Variables that had high correlation (rho>0.5) with AHI were excluded from the multiple logistic regression. Subsequently, a multivariable logistic regression model was used to assess the association of all potential predictors to DMV. Variables with a *p*-value < 0.05 were established as independent predictors. The adjusted association between AHI and DMV was explored using multiple logistic regression adjusting for age, gender, BMI, bearded, no teeth, Mallampati classification, thyromental distance, ability to extend lower jaw, and alcoholism. The adjusted odds ratio and 95% confidence intervals were reported.

All statistical analysis was conducted using the Statistical Analysis System (SAS) statistical software package (version 9.04.01 SAS Institute Inc, Cary, NC, USA.)

## Results

**Fig. 1:**
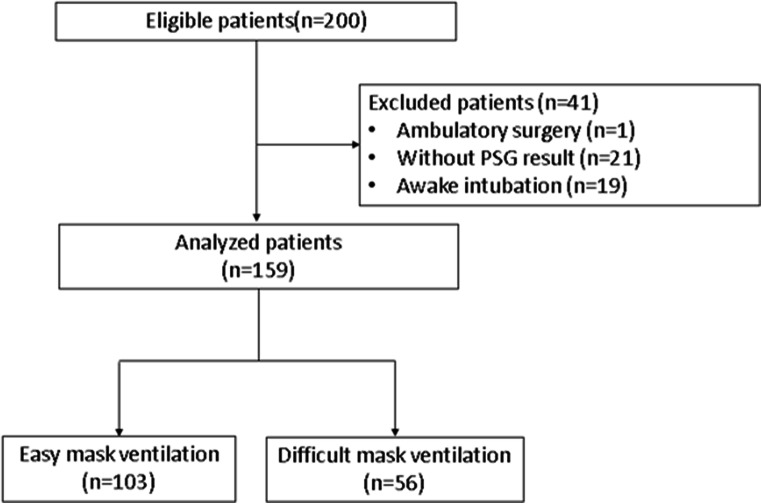
Patient enrollment flow diagram

**Fig. 2:**
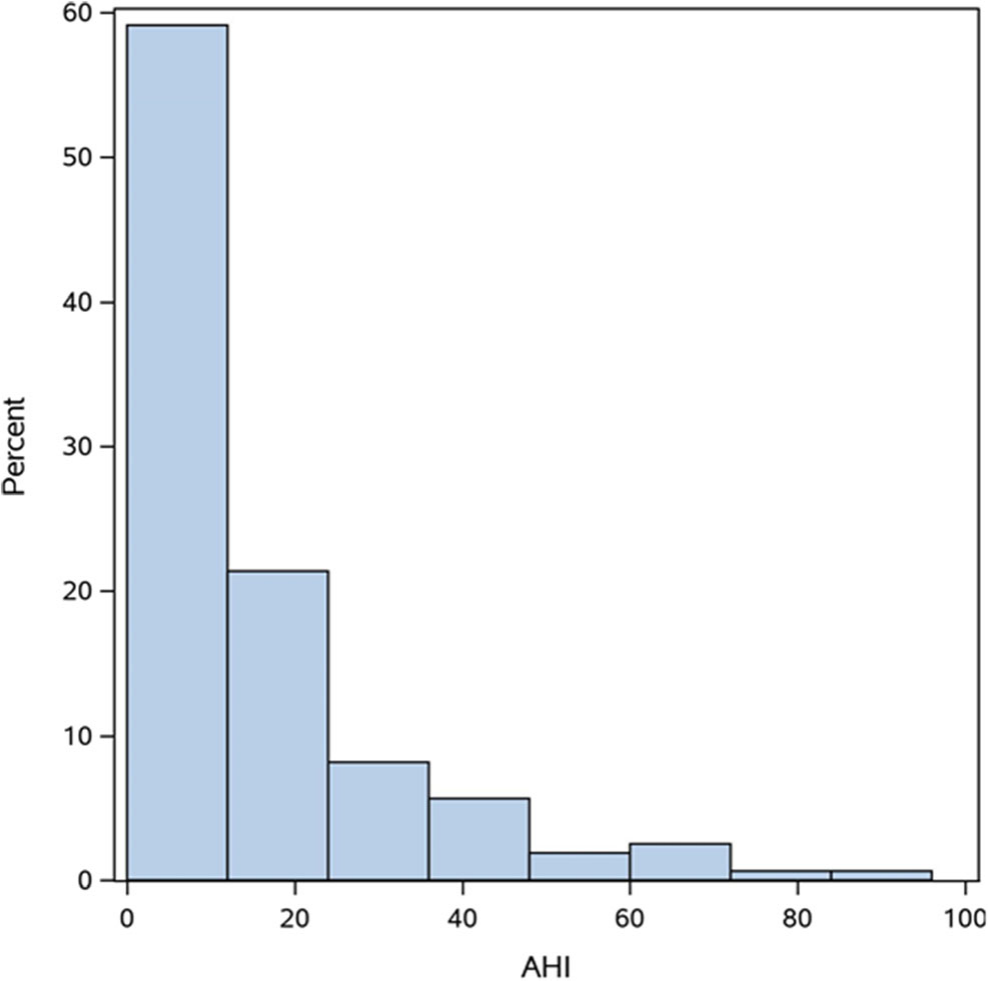
Histogram of apnea-hypopnea index (AHI)

**Fig. 3:**
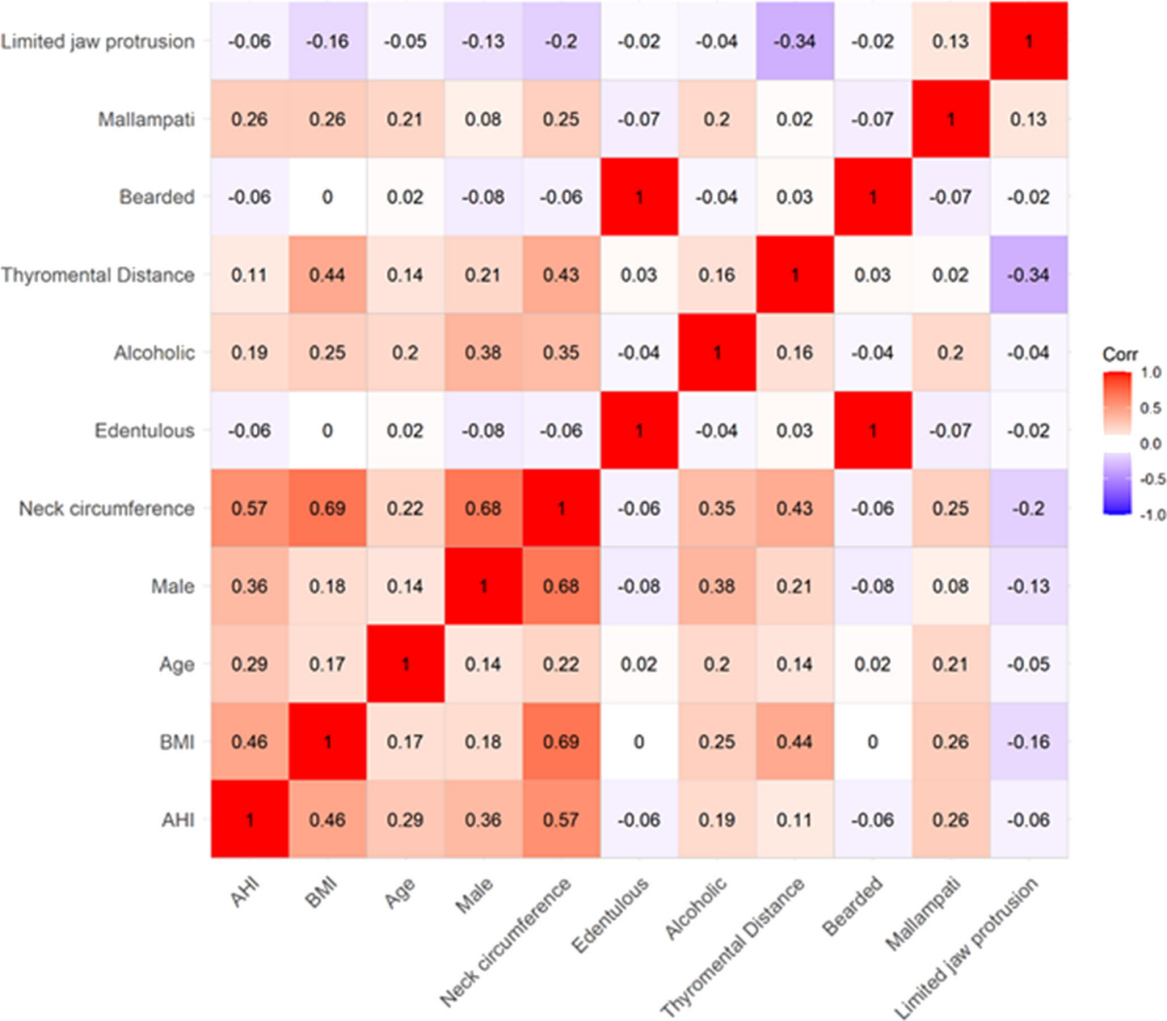
Pair-wise Pearson correlation coefficients between AHI and other variables, including age, male, BMI, neck circumference, modified Mallampati classification, thyromental distance, ability to extend lower jaw, and alcoholism

In this prospective cohort study, a total of 200 cases initially met our inclusion criteria. After excluding cases with missing AHI and inability to do mask ventilation, 159 cases were included in the final analysis (Fig. 1). Patients were aged 18 years or older who underwent elective surgery, with ASA I-III, and general anesthesia. Perioperative baseline characteristics and demographics are shown in Table 1. The median AHI was 10.9 [0.0, 95]. The histogram of AHI is shown in Fig. 2. The primary outcome showed that the unadjusted odds ratio (OR) of DMV related with a 5 unit increase of AHI was 1.50 [1.29, 1.76], *p*< 0.001. For the secondary outcome, gender, Mallampati classification, thyromental distance, ability to extend lower jaw, and alcoholism were all put in the multivariable logistic regression model. It showed that AHI, age and Mallampati classification were independent factors of DMV. Among these, AHI was highly related to DMV (adjusted OR of DMV with a 5 unit increase of AHI, 1.28 95% CI 1.08–1.52). Neck circumference was found to correlate with AHI (correlation coefficient of 0.57), and was therefore excluded from the model. In the multivariable analysis, AHI, age, and Mallampati classification continued to maintain their statistical significance (*p*<0.05), as independent factors (Table 2). Meanwhile, AHI was related to BMI (*p*<0.0001), age (*p*=0.0282), gender (*p*<0.0001), neck circumference (*p*<0.0001), alcoholism (*p*=0.0495), thyromental distance (*p*=0.0022), and Mallampati classification (*p*=0.0003). The correlation between AHI and other variables is shown in Fig. 3. Due to the lack of statistical desirable sample size of patients with beards (*n*=1) and no teeth (*n*=1) in the study population, these two factors were not assessed in the final analysis.

**Table 1:** Baseline characteristics by group

Factor	Total (*N*=159)	Easy ventilation (*N*=103)	Difficult ventilation (*N*=56)	ASD
Age (years)	38 ± 13	34 ± 13	45 ± 10	0.88
BMI (kg/m^2^)	25 ± 4.7	24 ± 4.3	28 ± 4.3	0.87
Male	82 (52)	40 (39)	42 (75)	0.78
Thyromental distance (cm)	6.0 ± 1.2	5.9 ± 1.2	6.3 ± 1.1	0.39
Neck circumference (cm)	37 ± 4.4	36 ± 3.5	40 ± 4.4	1.07
AHI (/h)	10.1 [3.8, 21]	6.3 [2.4, 13]	20.7 [11, 37]	1.31
Bearded	1 (0.6)	1 (1)	0 (0.0)	0.14
Edentulous	1 (0.6)	1 (1)	0 (0.0)	0.14
Alcoholism	28 (18)	9 (8.7)	19 (34)	0.65
Radiotherapy	0 (0.0)	0 (0.0)	0 (0.0)	
Snoring history	120 (76)	67 (65)	53 (95)	0.79
Limited jaw protrusion				0.33
1	144 (91)	90 (87)	54 (96)	
2	14 (8.8)	12 (12)	2 (3.6)	
3	1 (0.63)	1 (0.97)	0 (0.0)	
Mallampati				0.79
1	77 (48)	62 (60)	15 (27)	
2	47 (30)	28 (27)	19 (34)	
3	24 (15)	8 (7.8)	16 (29)	
4	11 (7)	5 (4.9)	6 (11)	

Statistics were summarized as mean ± SD, median [Q1, Q3], or *N* (%)

**Table 2:** Multivariable logistic regression model

	Estimated OR (95% CI)	*p*-value
AHI (5 unit)	1.28 [1.08, 1.52]	<0.01*
Age (years)	1.05 [1.01, 1.09]	<0.01*
Male	2.57 [0.94, 7.06]	0.07
BMI (kg/m^2^)	1.12 [0.99, 1.26]	0.08
Mallampti classification	1.76 [1.04, 2.96]	0.03*
Thyromental distance (cm)	0.99 [0.66, 1.50]	0.97
Limited jaw protrusion	0.18 [0.02, 1.52]	0.12
Alcoholism	2.16 [0.68, 6.89]	0.19

AHI is calculated as the sum of all apneas and hypopneas, divided by total hours of sleep time

*OR*: odds ratio; *CI*: confidence interval

*AHI, age, and Mallampti classification were statistical significant factors (*p*<0.05)

The estimated OR was estimated using multivariable logistic regression model.

## Discussion

This study was designed to determine the association between AHI and DMV. We found that with the 5-unit increase of AHI, the incidence of DMV was significantly higher. In this high-risk population, 31% had AHI≤5. In the remaining patients, values typically ranged from 5 to 60/h. Therefore, a patient with 60/h has about a 15 times increase in the odds of difficult ventilation comparing to a patient with 5/h. The secondary outcome showed that AHI, age, and Mallampati classification were independent factors of DMV. Indeed, AHI was shown to be highly related to DMV. Normally, the most widely used definition of DMV was by Han et al. [17], which was described as “inadequate, unstable, or requires two providers” with or without muscle relaxant. Impossible mask ventilation was defined as the inability to mask ventilate with or without muscle relaxant. In this study, we rated mask ventilation according to Langeron et al. [3] as “the inability of an unassisted anesthesiologist to maintain oxygen saturation >92%, as measured by pulse oximetry, or to prevent or reverse signs of inadequate ventilation during positive-pressure mask ventilation under general anesthesia.” As Sato et al. [18] mentioned, one-hand mask ventilation which was categorized into the easy group in our study was unsuitable for patients with obesity and severe sleep–disordered breathing. It was also proved in our study as well that AHI was 20.7 [11, 37]/h and BMI was 28 ± 4.3 kg/m^2^ in the difficult group.

Numerous previous studies on the prediction and treatment of DMV (difficult mask ventilation) concluded that age>55, BMI >26 kg/m^2^, presence of beard, no teeth, and snoring are 5 independent factors related to DMV [3]. In addition, neck radiotherapy, neck circumference, male gender, Mallampati grade, and severely limited jaw protrusion are known as the risk factors for DMV [2, 4, 10]. Among these studies, one of the independent factors of DMV is a history of snoring (patients were asked if they were habitual snorers or not) [3]. Although OSA is very common [19], the majority of patients presenting to the operating room with OSA have not previously been diagnosed and lacked self-awareness of this condition. In this study, all patients underwent PSG preoperatively, which could provide detailed diagnoses of OSA and validate the accuracy of patient’s self-reported history of snoring/choking for air during sleep. In conclusion, the PSG result was clinically more reliable than a declared history of snoring, as provided by the patients.

From a previous study, higher age, higher BMI, greater neck circumference, male gender, and higher Mallampati score were significantly correlated with a high risk of OSA [20]. Concurrently, these overlapping risk factors were also related to DMV [2–4, 10]. It was found that patients with OSA had a three- to four-fold higher risk of difficult intubation or mask ventilation, or both, compared to non-sleep apnea patients [21]. However, most previous studies used diagnosed OSA as the primary outcome. There was also a study found that difficult mask ventilation was predictive of undiagnosed OSA [22]. While PSG is the diagnostic standard of OSA [11], one of its important parameters is AHI, which is calculated by adding all apneas and hypopneas and then dividing by total sleep time. An AHI of 15 or more events per hour, or five or more events per hour in the presence of symptoms or cardiovascular comorbidities, is the diagnostic criteria for OSA [11, 12]. In this study, AHI was used as a new continuous measurement for DMV that provided more information on the simple presence or absence of diagnosed OSA.

Normally, a conventional PSG examination is time-consuming and complicated to perform, requiring a minimum of 22 wires attached to the patient. With the development of home sleep testing devices, patients are more likely to accept and enjoy certain conveniences, as in this study. The portable PSG consisted of continuous recording with three sensors (respiratory effort belt, cannula, and non-invasive pulse oximetry). Among the 200 patients, only 21 patients did not have the PSG report due to inadequate time of measuring and feeling discomfort during the PSG testing. Concurrently, other parameters such as oxygen desaturation and heart rate during the night might further aid decision-making for anesthesiologists.

As for the Chinese population, there was a limited number of articles on mask ventilation. Relatively, most of the trials were from the European and US populations. Furthermore, there were fewer bearded Chinese patients to consider, as well was differences in head and facial structures.

For the secondary outcome, age, AHI, and Mallampati score were three significant, independent factors for the DMV. Most DMV evaluations consist of questionnaires which are time consuming for anesthesiologists to complete and score [13]. Additionally, it is unclear which predictive method is easier to perform and reliable for use in clinical settings. In our study, we demonstrated a possible solution that efficiently utilizes portable PSG, which, combined with AHI outputs, age, and Mallampati scores, can a predict patient’s risk of DMV. Moreover, these three factors remained objective compared to the subjective answers extracted from questionnaires. Certain predictive models mutually benefit both the patient and anesthesiologist by saving time, avoiding recall bias, raising patient’s satisfaction score, and providing a pre-liminary assessment of potential OSA in otherwise undiagnosed patients.

One limitation of the study was the relatively small sample size (*n*=159). To explore further applications of AHI in DMV patients, we will need to study larger patient populations. Also, our study focused on Chinese patients, which are ethnically different from other global populations. A larger, more versatile sample would be desirable for further study to demonstrate the effectiveness of our predictive model. Another limitation was that in this study, AHI was accomplished with polygraphy which was insufficient to rule out OSA comparing to traditional PSG [23]. Meanwhile, some of the patients reported an uncomfortable experience while they were sleeping during the PSG testing. Future models of PSG equipment should be developed to improve certain concerns from patient’s perspective.

## Conclusion

Elevated AHI is associated with increased risk of DMV. AHI, age, and Mallampati classification were independent factors of DMV in the Chinese population, and could altogether serve as a convenient and objective predictive model for DMV.

## Data Availability

De-identified data is available for investigators. Requests should be addressed to iriswjy1991@gmail.com.
